# Opioid-Induced Psychosis in a Patient With Sickle Cell Disease

**DOI:** 10.7759/cureus.15557

**Published:** 2021-06-09

**Authors:** Terence Tumenta, Amod Thanju, Pradilka Perera, Jisha Kallikkadan, Patrice Fouron, Tolulope Olupona

**Affiliations:** 1 Psychiatry, Interfaith Medical Center, Brooklyn, USA

**Keywords:** opioid, hydromorphone, aggression, psychosis, sickle cell disease

## Abstract

Sickle cell disease (SCD) is a common inherited hemoglobin disorder in which people have atypical hemoglobin, known as hemoglobin S. It is highly prevalent in non-Hispanic Blacks and people of Arab descent. It causes a distortion of the shape of red blood cells, leading to occlusion of blood vessels and thus tissue hypoxia and injury. The resultant infarction/reperfusion, in turn, causes fatigue and pain. Patients with SCD require constant analgesic medications for pain management. In the general population, opioids are amongst the most prescribed medications for pain management and the trend has been gradually growing during the past two decades. Side effects commonly associated with opioids are gastrointestinal and central nervous system-related, with up to 80% of patients experiencing at least one adverse effect.

We report the case of a 36-year-old male patient who has a history of cannabis use and no prior psychiatric history, who developed acute psychosis while receiving a high dose of hydromorphone for sickle cell pain crisis. This case contributes to the growing literature about opioid-induced psychosis and also explores psychosis in sickle cell disease. Understanding the pharmacology and potential side effects of opioids is critical given the increasing number of patients using prescribed and illicit opioids.

## Introduction

Sickle cell disease (SCD) is a common inherited hemoglobin disorder in which people have atypical hemoglobin also known as hemoglobin S that is responsible for distorting the shape of red blood cells which then occlude vessels to cause pain, commonly known as sickle cell crisis. Vaso-occlusion leads to tissue hypoxia and injury, via infarction/reperfusion. This pathophysiological characteristic of SCD not only causes excruciating pain but also causes significant lifelong morbidities, such as hemolysis, vaso-occlusive crisis, acute chest syndrome, and multi organ failure [1,2]. For this reason, SCD is a major health problem that requires constant analgesic medications [3]. While mild to moderate pain is managed with nonsteroidal anti-inflammatory drugs (NSAIDs), for persistent pain, depending on its severity, an opioid is required [4]. Opioid analgesics are controlled substances that have a high risk for developing tolerance, dependence, and addiction. According to the Centers for Disease Control and Prevention, physicians can only recommend opioid treatment if the desired benefits outweigh the risks in terms of pain and function [5]. Opioid analgesics are considered an appropriate therapy for acute or chronic persistent pain in SCD patients [3]. The mu, kappa, and delta-opioid receptors mediate analgesia spinally and supraspinally [6]. Hydromorphone is an opioid analgesic that is commonly indicated in moderate to severe acute pain or severe chronic pain. It has very similar pharmacological properties compared with morphine, yet it is two to eight times more potent than morphine [5,6]. Additionally, hydromorphone is a mu-opioid receptor agonist with weak agonistic properties at the delta and kappa-opioid receptors. There is converging evidence suggesting dopaminergic modulation via the mu and kappa-opioid receptor pathways [7-9], and that dysregulation of dopaminergic activity in the brain is responsible for psychosis [10]. However, the association between opioid use and psychosis has not been clearly understood. Evidence in literature has shown a common association of psychosis with methamphetamine, amphetamine, cannabis, cocaine, alcohol, lysergic acid diethylamide (LSD), phencyclidine (PCP), ecstasy, and ketamine [11]. However, there is unclear and minimal evidence about opioid-induced psychosis.

We present the case of a 36-year-old male with no prior psychiatric history, who developed acute psychosis in the hospital after receiving high doses of hydromorphone for sickle cell pain crisis, over a three-day period. This case is unique in the time sequence of development and resolution of the patient’s symptoms with a dose reduction and eventual cessation of hydromorphone use. He did not require scheduled antipsychotic medications and was free of symptoms at follow-up, 30 days after discharge.

## Case presentation

A 36-year-old male patient with no prior past psychiatric history, was admitted to the hospital in the context of sickle cell pain crisis. His past medical history was significant for SCD (HbSS), with complications including priapism, deep venous thrombosis, and pulmonary embolism. His past surgical history included penile implant and cholecystectomy. His home medication regimen was hydromorphone 4mg by mouth every four hours as needed; hydroxyurea 500mg by mouth twice daily; cyclobenzaprine 5mg by mouth three times a day; apixaban 5mg by mouth twice daily; gabapentin 400mg by mouth three times a day; oxycodone/paracetamol 10/325mg by mouth every four hours; folic acid; and multivitamins. On admission, routine laboratory tests were clinically significant for a hemoglobin level of 7.3 g/dl, leukocytosis at 18,600/ul, and low vitamin D level at 5.9L. His urine toxicology screen was positive for cannabis and opiates.

Upon admission, patient received 8mg of hydromorphone intramuscularly (IM) in two divided doses of 4mg each, five hours apart. He was then placed on hydromorphone 4mg IM every four hours as needed, receiving an additional 8mg over eight hours, making a total of 16mg of hydromorphone in less than 15 hours. However, the patient complained of persistent pain and hydromorphone was adjusted to 6mg IM every three hours. He was given a total of 48mg of hydromorphone every 24 hours thereon until the third day of admission. In total, the patient received 160mg of hydromorphone in 85 hours 15 minutes (3.55 days). On the third day of hospitalization, the patient was reportedly intrusive and hostile towards his roommate. He physically attacked his roommate unprovoked. Psychiatry was consulted as the patient was reportedly talking to himself and stating that his roommate was playing with his mind.

On psychiatric evaluation, patient was preoccupied with his relationship with his girlfriend and exhibited persecutory delusions about people trying to hurt him. He reported that his girlfriend and her family were stealing his property, trying to get him through the hospital phones, and said that they came to the hospital to hurt him. Capgras delusion was elicited as patient believed that his roommate impersonated his girlfriend and he repeatedly asked staff to check the monitoring cameras. He denied auditory, visual, and tactile hallucinations. His thought process was linear, logical, and goal-directed, though he perseverated about his girlfriend trying to hurt him. He denied suicidal or homicidal ideation, intent, or plan.

The team considered the possibility of delirium. However, the Confusion Assessment Method score was negative, and he was fully alert and oriented. Based on patient’s history, the high dose of hydromorphone, the time sequence of his symptomatology, his mental status examination, and a Naranjo score of 7, a diagnosis of hydromorphone-induced psychosis was considered. He was given haloperidol 5mg intramuscularly to prevent further agitation. Hydromorphone was reduced to 2mg by mouth every four hours as needed and he was started on oxycodone 300mg by mouth every four hours as needed, for pain management.

The next day, the patient was re-evaluated by the psychiatry team. He was still irritable and continued to express themes of mistrust and persecutory delusions of being followed and threatened by his girlfriend and her family. He received haloperidol 5mg IM for agitation and another 5mg by mouth after 12 hours.

Over the next 48 hours, he became less agitated, and his delusions were improving. Hydromorphone was discontinued. Three days after the onset of his symptoms, oxycodone was down titrated to 20mg by mouth every four hours as needed. His persecutory delusions continued to improve until discharge.

## Discussion

The Diagnostic and Statistical Manual, 5th edition (DSM 5), defines substance/medication-induced psychotic disorder as delusions and/or hallucinations related to the physiological effects of a substance or medication, based on evidence from the history, physical examination, or laboratory findings [11]. It emphasizes that delusions and/or hallucinations must develop during or soon after substance intoxication or withdrawal or after exposure to a medication, or that the involved substance/medication can produce delusions/hallucinations.

There is literature and evidence pointing to a link between cannabis and psychosis, with prospective, longitudinal, and epidemiological studies all indicating a link between cannabis use and schizophrenia [12]. According to our case presentation, the patient had no prior psychiatric history. Furthermore, despite the patient's history of regular cannabis use, there was no previous reported or documented history of psychotic symptoms. Other possible causes of psychosis in our patient included sickle cell disease and delirium. There has been an increase in the number of reports of psychosis associated with sickle cell disease [13,14]. At the moment, the pathophysiology is unknown, and more research is required. Sickle cell disease has also been linked to behavioral and emotional issues [15]. Delirium should always be considered in such patients due to reports of an increased risk, though the risk appears to be lower with hydromorphone compared to other opioids [16].

There is little research on opioid-induced psychosis, and it is mostly limited to case reports [17]. Sivanesan et al. conducted a review of the literature on opioid-induced hallucinations and concluded that, while uncommon, opioid-induced hallucinations are likely underreported due to the tolerable intensity of many hallucinations and fear of being labeled as psychiatrically unstable [18]. 

It is critical to identify patients with substance-induced psychotic disorder as soon as possible in order to provide timely and effective treatment, as well as follow-up visits. According to Alderson et al., the 15.5-year cumulative hazard rate for a diagnosis of schizophrenia after a diagnosis of substance-induced psychotic disorder is 17.3% [19]. They reported that those who developed schizophrenia required more than two years of follow-up, highlighting the importance of psychiatric follow-up after an initial episode.

The major weakness is that this is a case report, which ranks at the lowest level of evidence and a cause-effect relationship cannot be proven. However, this case contributes to the evolving literature on the role of opioid receptors in the development of psychosis.

## Conclusions

The effects of substances can be classified as either pro-psychotic or antipsychotic. Though there is evidence suggesting a link between certain substances and psychosis, the link with opiates is less clear at the moment. Given the probable risk of psychosis associated with opioids on the one hand, and the risk associated with sickle cell disease on the other, large doses of hydromorphone should be used with caution in patients with sickle cell disease.
